# Supplementary material for: Tuning Magnetic Properties of a Carbon Nanotube-Lanthanide Hybrid Molecular Complex through Controlled Functionalization
Source: Molecules. 2021 Jan 22;26(3):563. doi: 10.3390/molecules26030563 (PMC7866014; doi:10.3390/molecules26030563)
Supplement: Supplementary file 1 [file molecules-26-00563-s001.pdf]

Tuning magnetic properties of carbon nanotube-lanthanide hybrid molecular complex through controlled functionalization

I. S. Mosse[⊥1], V. R. Sodisetti[⊥1], C. Coleman[⊥1], S. Ncube[1], A. S. de Sousa[1], R. M. Erasmus[2], E. Flahaut[3], T. Blon[4], B. Lassagne[4], T. Šamořil[5] and S. Bhattacharyya[*1,2]

[1]Nano-scale Transport Physics Laboratory, School of Physics, University of the Witwatersrand, Johannesburg Wits 2050, South Africa.
[2]DST-NRF Centre of Excellence in Strong Materials and School of Physics, University of the Witwatersrand, Johannesburg, Wits 2050, South Africa.
[3]CIRIMAT, Université de Toulouse, CNRS, INPT, UPS, UMR CNRS-UPS-INP No. 5085, Université Toulouse Paul Sabatier, Bât. CIRIMAT, 118, route de Narbonne, 31062, Toulouse, cedex 9, France.
[4]LPCNO, Institut National des Sciences Appliquées, 135 avenue de Rangueil, 31077 Toulouse Cedex 4 – France.
[5]TESCAN Orsay Holding, a.s., Libušina tř. 21, 623 00 Brno, Czech Republic.

Supporting Information

1. CNT functionalization analysis

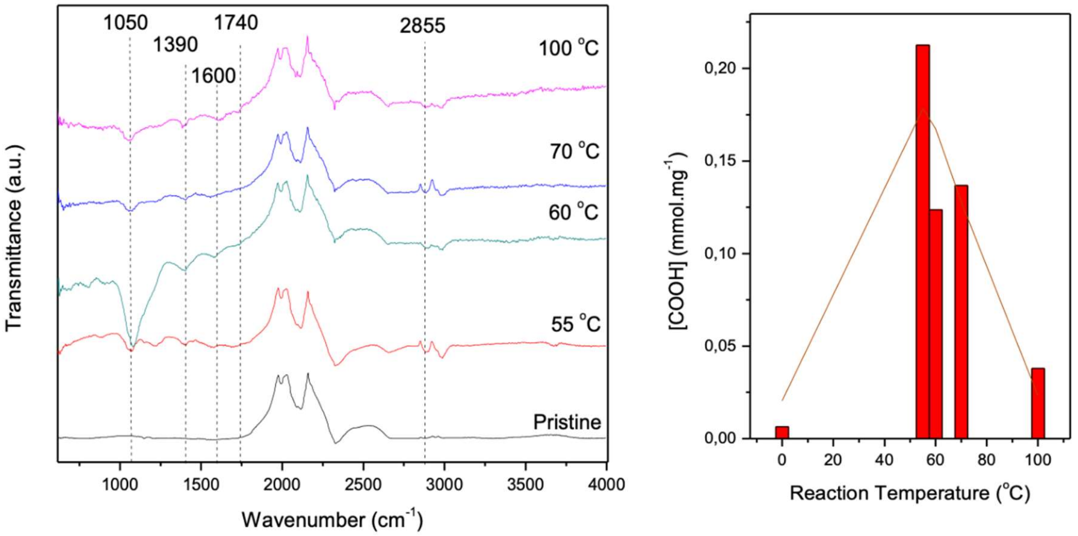

Figure S1: CNT Functionalisation analysis: a) FT-IR spectra of MWNTs treated at varied oxidative temperatures showing characteristic -COOH peak at ~1700 cm^{-1} wavenumber . b) Boehm Titration results of carboxylic groups present on acid treated carbon nanotubes with varied temperature conditions.

Fourier-transform infrared spectroscopy (FT-IR) is a complementary technique in evaluating the functional groups (-COOH) on the nanotube. FT-IR spectra of pristine and functionalized MWNTs treated at various temperatures (55 °C, 60 °C, 70 °C, and 100 °C) were collected and find the appearance of additional peak at ~ 1720 cm^{-1} in the absorbance spectrum associated with C=O stretching frequency [1] for the functionalized MWNTs (figure S1 a). This additional peak can be attributed to carboxyl groups (-COOH) formed during the oxidation of MWNTs.

⊥ These authors contributed equally towards this work
*Correspondence should be addressed to somnath.bhattacharyya@wits.ac.za

Further to quantify the number of carboxyl groups present on the nanotube, Boehm titration was performed on the functionalized MWNTs. The titration results reveal that high number of carboxylic group present in the CNT sample treated at 55 °C (0.237 mmol COOH/mg CNT) and low number of carboxylic groups in the sample treated at 100 °C (0.038 mmol COOH/mg CNT). However, from the literature it is envisioned that acid treatment at higher temperatures would result -COOH dimer formation (-$C_2H_2O_4$ - Oxalic functional groups) [2].

2. Raman Spectroscopy - G band position

Figure S2 provides information on the shift of G peak position in the MWNTs treated with functionalized conditions temperature and time respectively, excited with 514.5 nm wavelength line source. G peak position has shifted to higher wave number for both the functionalization conditions used in this study. It's well established in the literature that the G peak shift to higher frequencies in the case of *p*-type doping [3] and shifts to lower frequencies in the case of n-type doping [4]. During the creation of oxygen functional groups an π-electron transfer from the MWNT to the oxygen atoms happens and this in turn can influence the G band shift towards higher frequencies [5]. In our study we have noticed that MWNTs treated with varied temperatures showed G band position at higher frequencies (1595 cm^{-1}) than that of MWNTs treated with extended oxidation times (1589 cm^{-1}). This further supports our new CFD analysis that more oxygen functional groups are formed on the CNTs treated with higher temperatures and this can be used to add more Gd ions on to the nanotube surface.

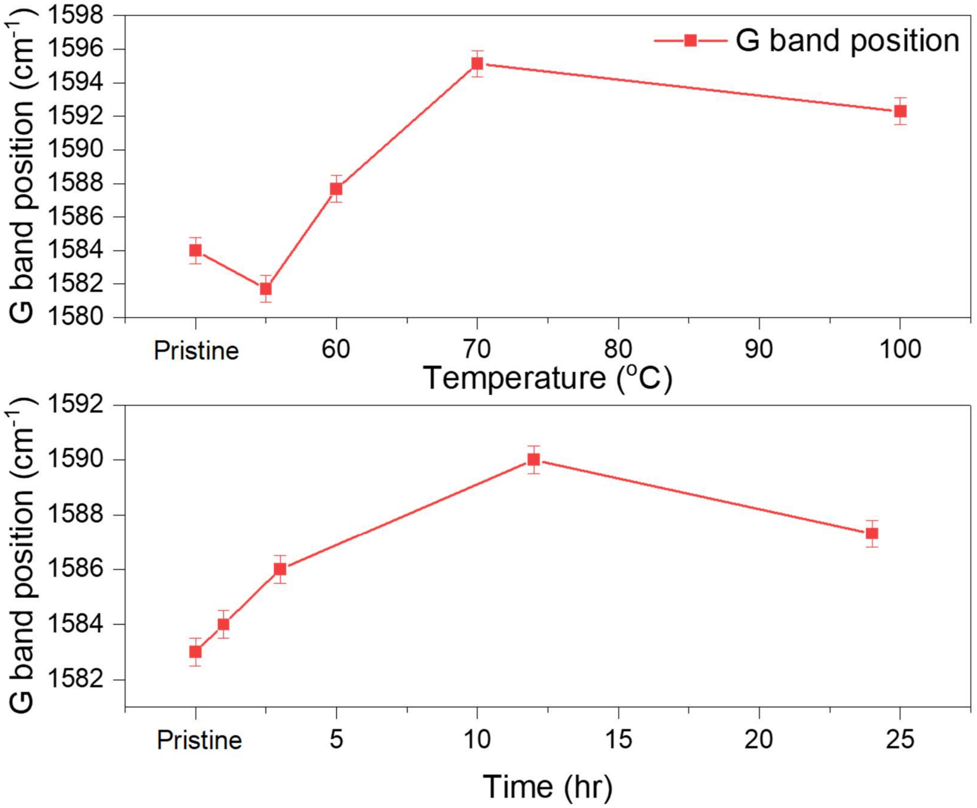

Figure S2. Change in G band position with respect to a) varied temperatures and b) extended oxidation times.

3. Susceptibility and inverse susceptibility Gd[DTPA]

By comparing the results of SQUID magnetometry for the various samples we are able to establish the origin and type of magnetic interaction. As can be seen in figure S3b, the pristine MWNTs show a very small paramagnetic response this has been observed before and is attributed to ferromagnetic particles remaining from synthesis. The effect of these particles are however small when compared to the magnetic response of the Gd functionalized nanotubes. In this study the molar susceptibility was used along with the Curie-Weiss law to determine the properties such as the Curie constant and Weiss temperature presented in the paper. This was done to allow for calculation of the effective moment which requires knowledge of molar concertation of the magnetic species. Figure S3(a) , the Gd-DTPA complex clearly showed paramagnetic susceptibility. The effective moment calculated from the inverse susceptibility of the Gd-DTPA was found to be 8.7 μ_B which is roughly 10 % larger than that of the bare Gd^{3+} ion theoretical value [6]. But smaller than the typical experimental values. Modification of the Gd effective moment, both enhancement and reduction, has been observed in Gd-carbon systems and is generally attributed to correlation effects [8] between Gd ion and carbon lattice or molecule. The Increase in moment here indicates a ferromagnetic coupling between Gd and DTPA molecule [7, 8, 9, 10]. The Gd-DTPA (Figure S3 a) which is paramagnetic differs significantly from the functionalized Gd-DTP-MWNT complex as presented in the paper, this is a strong indication that the resultant interaction observed is due to MWNT-Gd correlations and not dominated exclusively by the Gd-DTPA. This does present some difficulty in determining the effective moment of the respective samples as the susceptibility is presented in terms of number of moles of only the Gd found in each sample. The concentration was determined from the elemental analysis. This means, as stated in the manuscript, that the effective moment calculated in this way will lead to an over estimation as it does not take into account the carbon nanotube contribution.

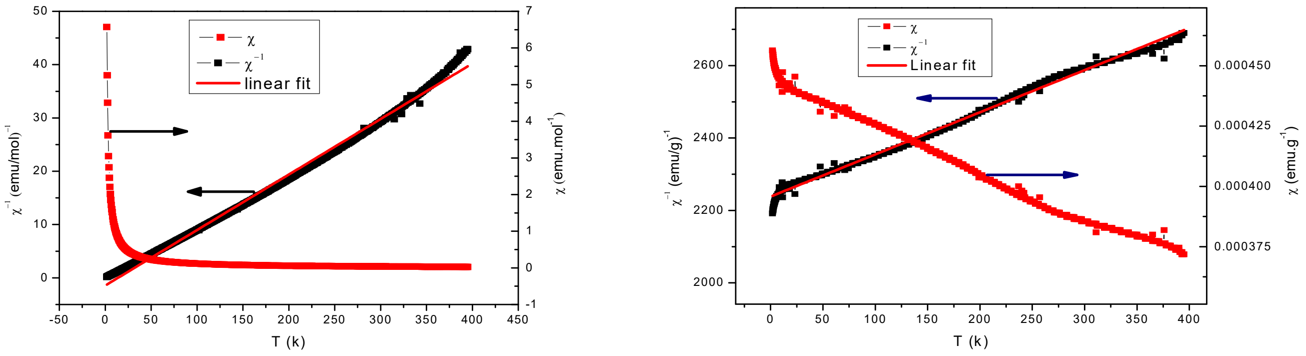

Figure S3. The plot of temperature dependence on susceptibility and inverse susceptibility for a) Gd-DTPA molecular complex and b) pristine MWNTs.

Reference:

1. Sahebian, S., Zebarjad, S.M., vahdati Khaki, J. *et al.* A study on the dependence of structure of multi-walled carbon nanotubes on acid treatment. *J Nanostruct Chem* 2015, **5,** 287–293.
2. Andrew, P. Wildman. The influence of Oxalic acid on rates of strong chelate exchange, Honors in Chemistry, Whitman College, Washington, WA 99362, USA, May 11, 2016.
3. Samsonidze, G., Saito, R., Jorio, A., Souza Filho, A.G., Pimmenta, M.A., Dresselhaus, G., and Dresselhaus, M.S. Phonon triagonal warping effect in graphite and carbon nanotubes. Phys. Rev. Lett. 2003, 90, 27403.
4. Thomsen, C., and Reich, S. *et al*. Double resonant Raman scattering in graphite. Phys. Rev. Lett. 2000,85,

5214.

5. Chakrapani, N., Curran, S., Wei, B. *et al*. Spectral fingerprinting of structural defects in plasma-treated carbon nanotubes. *Journal of Material Research*, 2003, 18, 2515-2521.
6. J. Li, T. Wang, Y. Feng, Y. Zhang, M. Zhen, C. Shu, L. Jiang, Y. Wangb and C. Wang, Dalton Trans., 2016, 45, 8696.
7. J. Zhang, Y. Ye, Y. Chen, C. Pregot, T. Li, S. Balasubramaniam, D. B. Hobart, Y. Zhang, S. Wi, R. M. Davis, L. A. Madsen, J. R. Morris, S. M. LaConte, G. T. Yee and H. C. Dorn J. Am. Chem. Soc. 2014, 136, 2630−2636.
8. R. Kitaura, H. Okimoto, H. Shinohara, T. Nakamura, and H. Osawa, Phys. Rev. B **76**, 172409
9. H. J. Huang and S. H. Yang, X. X. Zhang, J. Phys. Chem. B 1999, 103, 5928-5932.
10. R. F. Sabirianov, W. N. Mei, J. Lu, Y. Gao, X. C. Zeng, R. D. Bolskar, P. Jeppson, N. Wu, A. N. Caruso and P. A. Dowben, J. Phys.: Condens. Matter, 2007, 19, 082201.
